# Transcriptome Analysis Reveals Novel Entry Mechanisms and a Central Role of SRC in Host Defense during High Multiplicity Mycobacterial Infection

**DOI:** 10.1371/journal.pone.0065128

**Published:** 2013-06-18

**Authors:** Jay Zhang

**Affiliations:** Genomics Research Centre, Griffith Health Institute, Gold Coast Campus, Griffith University, Southport, Queensland, Australia; University of Delhi, India

## Abstract

*Mycobacterium tuberculosis* (MTB) infects an estimated one-third of the global population and is one of the main causes of mortality from an infectious agent. The characteristics of macrophages challenged by MTB with a high multiplicity of infection (MOI), which mimics both clinical disseminated infection and granuloma formation, are distinct from macrophages challenged with a low MOI. To better understand the cross talk between macrophage host cells and mycobacteria, we compared the transcription patterns of mouse macrophages infected with bacille Calmette-Guérin, H37Ra and *M. smegmatis*. Attention was focused on the changes in the abundance of transcripts related to immune system function. From the results of a transcriptome profiling study with a high mycobacterial MOI, we defined a pathogen-specific host gene expression pattern. The present study suggests that two integrins, ITGA5 and ITGAV, are novel cell surface receptors mediating mycobacterium entry into macrophages challenged with high MOI. Our results indicate that SRC likely plays a central role in regulating multiple unique signaling pathways activated by MTB infection. The integrated results increase our understanding of the molecular networks behind the host innate immune response and identify important targets that might be useful for the development of tuberculosis therapy.

## Introduction

As a primary response to *Mycobacterium tuberculosis* (MTB) infection, activated macrophages increase the expression of a panel of phagocytic receptors, termed pattern recognition receptors (PRRs), which regulate inflammation-mediated microbial clearance [1]. Such receptors include mannose receptor, Toll-like receptors (TLRs), NOD-like receptors (NLRs), and complement receptors [2]. Activated PRRs activate cytoplasmic tyrosine kinase SRC, which has been linked to many intracellular signaling pathways in macrophages. Activated SRC is known to be involved in regulating many downstream inflammatory signaling pathways [3], [4].

Several gene expression profiling studies comparing pathogenic and non-pathogenic mycobacterial infection under relatively low multiplicity of infection (MOI) (MOI < = 10) have revealed significant differences in the expression of genes involved in a wide range of processes [5], [6]. In addition, different response patterns are also seen at different MOIs. Macrophage apoptosis depends, in part, on intracellular bacillary load, and rapid cytotoxicity occurs when a MOI threshold of 25 is exceeded [7].

We hypothesized that high intracellular loads of mycobacterial exposure would generate a disease-relevant gene expression profile. A system-wide analysis of these profiles would yield clinically specific pathways for diseases [8] and avenues for drug development. Our present study suggests the integrins (ITGA5 and ITGAV) as possible novel PRRs for mycobacterium entry into macrophages. We also reveal that SRC plays a central role in the host defense network. The host targets identified could be sound candidates for host-directed anti-mycobacterial therapies.

## Materials and Methods

### Cells, Cultures, and Media

The murine macrophage cell line J774A.1 (American Type Culture Collection, ATCC) was used in this study. J774A.1 cells were cultured in DMEM (HyClone from Thermo Scientific) medium containing 10% (v/v) fetal calf serum, 50 µg/ml of penicillin/streptomycin and 2 mM glutamine. Cells were used to conduct experiments when they reached ∼70% confluence. All treatments were performed in serum-free medium.

All mycobacteria were grown on Middlebrook 7H11 agar at 37°C, 5% CO2-95% air atmosphere. For broth cultures, H37Ra (ATCC 25177, the lab strain of MTB) and bacillus Calmette-Guérin (ATCC 35734, BCG, the vaccine strain of *M. Bovis*) were grown in 7H9 medium supplemented with glycerol (0.5%, vol/vol) and OADC supplement. *M. smegmatis* (ATCC 700084, mc^2^-155,) was grown in 7H9 medium supplemented with glycerol (0.5%, vol/vol) and ADS supplement. All liquid cultures were supplemented with 0.05% Tween 80.

### Macrophage Infections

In order to obtain a single cell suspension for an infection assay, the following procedure was performed as previously described [54]. Briefly, bacteria were centrifuged and washed twice in PBS, re-suspended in media (no additives), and sonicated at 30% power for 10 sec in a cuphorn sonicator, twice. Sonicated bacteria were dispersed by aspiration five times each with a 24-gauge needle, followed by an additional dispersion 5 times through a 30-gauge needle. This was then vortexed until no bacterial clumps were detectable, and the dispersed bacteria were allowed to stand for 5 min. The upper half of the suspension was then used for the experiments. Quantification of bacteria was done by taking absorbance at a 600-nm wavelength (0.6 OD corresponds to∼100×10^6^ bacteria). Cells were infected with mycobacterium species at a multiplicity of infection (MOI) of 50 in antibiotic-free DMEM (HyClone) 37C for 2 hours and then washed 3 times with fresh media to remove extracellular bacteria and further incubated for an additional 2 hours in DMEM. After the infection, cells grown on cover slips infected with various mycobacteria were stained using TB Quick Stain Kit (BD Diagnostic Systems). Twenty randomly-infected mouse macrophage cells were counted for intracellular bacterial load as well as infection rate under a Nikon microscope.

### RNA Isolation and Microarray Experiments

Total RNA was isolated from 2×10^6^ J774A.1 cells 4 hours after infection with various mycobacterial species and from un-infected cells. Total RNA was isolated using TRIzol reagent (Invitrogen, Carlsbad, CA, USA) following the manufacturer's protocol, followed by on-column digestion of DNA using the RNeasy Mini Kit (Qiagen, Valencia, CA, USA). RNA quantity and quality were assessed with a Qubit RNA Assay Kit using a Qubit 2.0 Fluorometer (Invitrogen, Carlsbad, CA, USA) and Agilent 2100 Bioanalyzer (Agilent, Santa Clara, CA, USA). 500 ng of total RNA was amplified using the GeneChip 3′ IVT Express Kit. Standard Affymetrix protocols were used to process and scan Affymetrix MOE430_2 microarrays (Affymetrix, Santa Clara, CA, USA).

### Microarray data analysis

Raw data from the probe sets for 39,000 transcripts was analyzed by Expression Console (Version 1.1, Affymetrix, Santa Clara, CA, USA) using global normalization to a target intensity of 200 by the MAS5 algorithm and afterwards eliminating probe sets with intensity values under 100. For genes with multiple probe sets, those containing the probe set suffixes _x_at, _s_at or _a_at, which possibly cross-hybridize to multiple transcripts, were removed. Probe sets with detection P-values greater than 0.05 (defined by the Affymetrix MAS5 algorithm) were removed. The remained list with fold-changes greater than 2.0 were calculated for each infection condition relative to the uninfected control. The microarray data has been deposited at GEO (Gene Expression Omnibus) with accession number GSE45675. Raw intensity values of these remaining 13142 probe sets were z-score normalized and unsupervised hierarchical clustering by Spotfire (TIBCO, Somerville, MA, USA) was applied to investigate potential underlying relationships between samples.

### Quantitative real time PCR and comparison with microarray data

Total RNA was used as a template for cDNA synthesis catalyzed by Superscript II (Invitrogen). Diluted cDNA from 50 ng total RNA was used as a template for real time reactions containing primer sets from PrimerBank [55] or designed by Primer 3 [56], and SYBR Green PCR Master Mix (Applied Biosystems) in accordance with the manufacturers' instructions. These reactions were carried out on an ABI 7900HT real-time PCR cycler (Applied Biosystems). GAPDH, 18S and beta-actin were used to normalize total RNA amounts per sample. The quantitative real time PCR data are presented as log2 transformed fold-change values. For log2 values between 1 and −1, which represent changes less than 2 fold-change, un-logged values were used. The primer pairs used in this study are listed in Table S7.

### Functional interpretation of microarray data with pathway and network analysis

Genes differentially expressed at least 2-fold up- or down- compared to uninfected control were imported and analyzed in IPA (Ingenuity Pathway Analysis, www.ingenuity.com) for global network interactions. Fisher's exact test was used to calculate a p-value determining the probability that each biological function assigned to that data set is due to chance alone. Canonical Pathway Analysis identified pathways with p-values≤0.05 from the IPA library that were considered significantly over-represented in the gene expression data. Ingenuity network analysis was used to display an interactive graphical representation of the interrelationships between molecules. Scores for IPA networks are the negative logarithm of the P-value, and indicates the likelihood of the genes analyzed in a network being found by random chance. Scores of 2 or higher have at least a 99% likelihood of not being generated by chance alone. For visual presentation, IPA Path Designer tool was used.

The genes that were altered by high MOI were functionally classified into biological processes using the PANTHER classification system (http://www.pantherdb.org). To assess the statistical enrichment of over-representation of these biological processes from our datasets relative to all mouse genes, a binomial statistic for multiple testing within the PANTHER system was applied [14].

## Results and Discussion

### Bacterial load of infected cells

Macrophages generated from human blood monocytes represent an excellent model to study host-pathogen interaction. However, practical and ethical concerns limit the applications of these cells. Rodent macrophages offer a suitable alternative for human macrophages, despite the genetic differences between them. Decades of studies suggest that the immune response in human and rodents shares a core profile of pathways. Therefore, it is not surprising that the mouse macrophage cell line, J774A.1, which provides high cell yields economically and with high reproducibility, has been widely used for early-stage drug discovery.

Gene expression analysis was examined in the murine macrophage cell line, J774A.1, challenged with BCG, H37Ra and *M. smeg (M. smegmatis)* for 4 hours. The infection rates of different mycobacterial species were >95%, and the average intracellular bacterial loads for BCG, H37Ra and *M. smeg* were 24, 25 and 29 per cell (range 10–52 per cell), respectively, with no significant difference among species. These results are similar to earlier studies in which the maximum load for intracellular bacilli per macrophage was about 25 [7], [9].

It has already been established that BCG and H37Ra, derived from the pathogenic mycobacterial species, *M. bovis* and MTB (H37Rv), respectively, are good non-BSL3 surrogates for MTB research and anti-tuberculosis discovery [10].With non-pathogenic *M. smeg* infection, macrophages are effective at killing most bacteria within 4 hours at low MOI, but the bacteria are able to stay alive and even grow for at least 12 hours at MOI >10 in surviving cells [11]. A previous study indicates that macrophages begin to undergo programmed death at 3 hours, most cells being killed by 20 hours after infection with BCG or H37Ra when the intracellular bacillary load exceeds a threshold of 25 [7]. Therefore, we decided to establish gene expression profiles of infected J774A.1 (MOI = 50) at 4 hours post-infection to maximize both the infection rate and macrophage survival.

### Global gene expression analysis in infected macrophages

**Figure 1:**
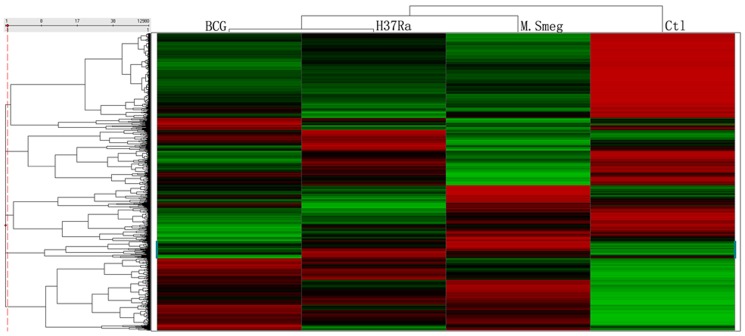
Unsupervised hierarchical clustering. A heat map displays z-score normalized and unsupervised hierarchical clustering for three infection conditions. The red and green color scale indicates higher and lower expression, compared with the median, across all conditions, respectively.

Unsupervised hierarchical clustering of probe sets filtered for detection above an arbitrary threshold demonstrates that macrophage response to BCG and H37Ra infection was distinct from that to *M.smeg* infection (Figure 1). Pathogenic BCG and H37Ra, belong to the same MTB complex and share greater sequence identity to each other [12] than to non-pathogenic species *M. smeg* [13].

**Figure 2:**
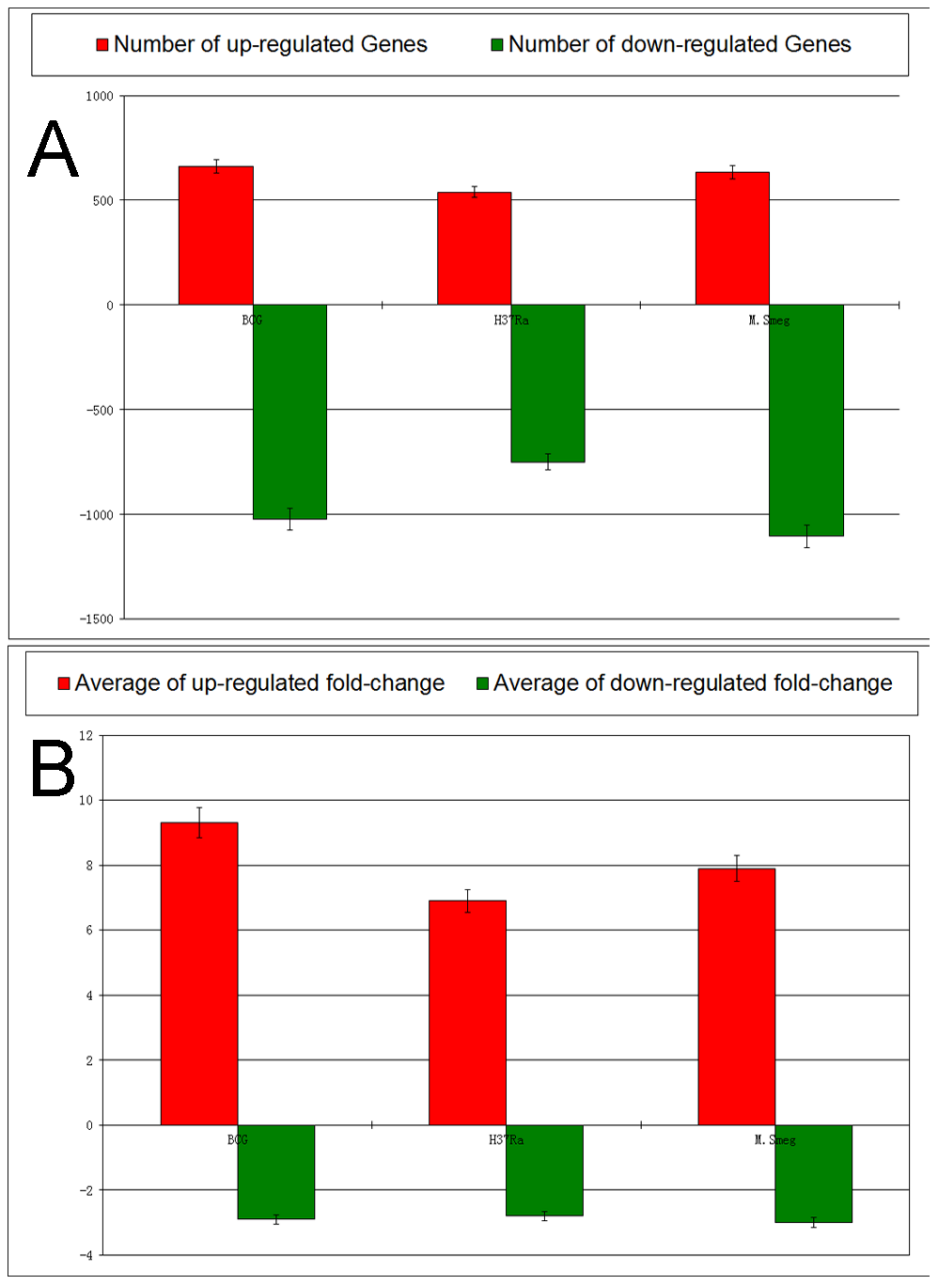
Comparison of numbers of differentially expressed genes and average fold changes. Shown are the number of differentially expressed genes and the average fold changes of regulated genes in the BCG, H37Ra, and *M. smeg*-challenged macrophages relative to the uninfected control. The height of the bars and the error bars represent the number of up-(red) or down-regulated (green) genes, the mean value of fold change from the up-(red) or down-regulated (green) genes, and 95% confident intervals.

Genes that were differentially-expressed by 2-fold compared with uninfected controls were then examined. Notably, the number of down-regulated genes exceeded the number of up-regulated genes in each mycobacteria-challenged group. However, the mean fold change in expression for the up-regulated genes was markedly greater than that for the down-regulated genes (Figure 2). Furthermore, the up-regulated genes were enriched in immune-related responses, intracellular signaling, and the cell communication. In contrast, cell cycle and metabolic processes, which are not directly related to macrophage immune functions, were enriched in the down-regulated genes [14] (Table S1). These findings highlight the complex nature of the interaction between the macrophage and the mycobacterium during infection. They are also consistent with previous findings showing that mycobacterial infection is associated with genome-wide immune-specific gene expression [15].

### Validation of regulated genes by quantitative PCR

**Figure 3:**
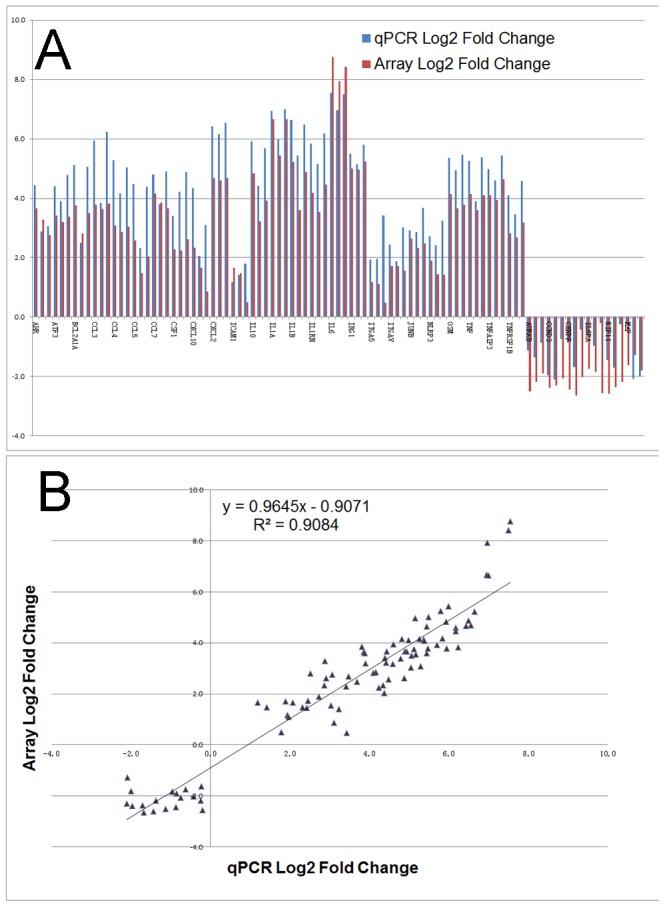
Validation of differential expression of a subset of genes by quantitative real-time PCR. (A) RT-PCR confirmation of Affymetrix array data on 31 selected up- or down-regulated genes. Log2 Q-PCR fold-change data are represented by blue bars, whereas log2 values from microarray experiments are depicted as red bars. (B) Correlation analysis of the microarray and Q-PCR transcript measurements for the same 31 regulated genes. The microarray log2 values were plotted against the Q-PCR log2 data. The correlation coefficient (R^2^) between the two analyses is 0.91. For details on gene abbreviations and fold change compared with control, see Table S2.

Thirty-one of the differential-expression changes were validated using quantitative real-time PCR (Q-PCR) in an independent sample set. A correlation plot showed a strong, positive association between QPCR and microarray data (R^2^ = 0.91) (Figure 3 and Table S2). This indicates that the microarray platform and the subsequent pathway analyses are robust compared with the sensitive, low-throughput Q-PCR approach.

### Pathogen-specific molecular pattern of host transcriptome

**Figure 4:**
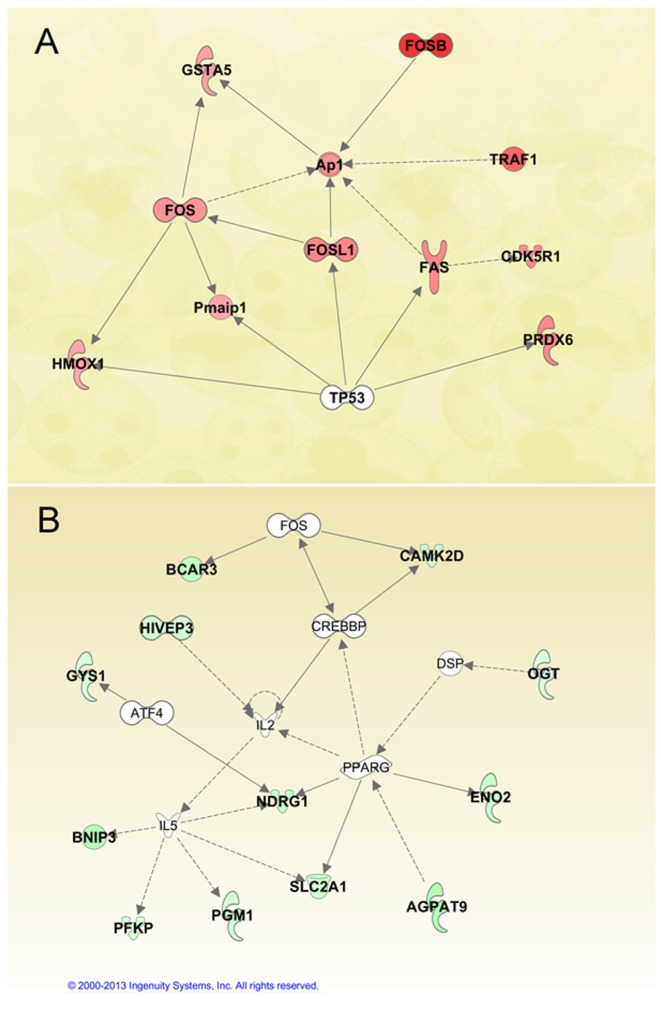
Pathogenic and non-pathogenic specific networks. Ingenuity Pathway Analysis (IPA) identified two top-scoring networks from genes with a ≥2 fold increase in expression in both BCG and H37Ra compared with M.smeg (4A, genes assigned a red fill), as well as the inverse comparison of M.smeg to both BCG and H37Ra (4B, genes assigned a green fill). Solid interconnecting lines show the genes that are directly connected and the dotted lines signify an indirect connection between the genes, with gene functions attributed by shapes assigned by the IPA knowledge base. Genes with no color are network members that are not differentially expressed. For details on gene abbreviations and the magnitude of expression changes, see Table 1.

To further explore infection-specific gene expression, we examined only genes that were up-regulated after infection. Genes that changed ≥2-fold in both of the pathogenic-derived mycobacterial species, BCG and H37Ra, compared with *M.smeg* (Table S3), were analyzed by pathway analysis. We examined two of the highest-scoring networks. Interestingly, for the pathogenic species (BCG and H37Ra), all of the genes were involved in apoptosis. FOS, FOSL1, GSTA5 and HMOX1 (HO-1) are key molecules involved in both the nuclear factor erythroid 2-related factor 2 (NRF2)-mediated oxidative stress response and aryl hydrocarbon receptor (AhR) signaling. The two pathways coordinately regulate phase I and II xenobiotic metabolism, and act as a cytoprotector with a role in anti-apoptosis for the host cells (Figure 4 and Table 1) [16]. However, most genes highly expressed in M.smeg are not involved in the immune response and apoptosis, but in metabolism (Figure 4 and Table 1).

**Table 1:** List of pathogen specific genes from figure 4.

Gene Symbol	BCG/M.Smeg	H37Ra/M.Smeg	BCG/Ctl	H37Ra/Ctl	M.Smeg/Ctl
Fosl1	2.8	3.9	23.1	32.3	8.3
Pmaip1	2	2.1	3.9	4.2	1.9
Gsta2	2.2	2.1	181.3	173.4	83.9
Fosb	4.7	2.9	6.5	4.1	1.4
Fos	2.4	2.2	2.2	1.9	−1.1
Cdk5r1	2.7	2.3	3.6	3.2	1.4
Prdx6	2.8	3.1	4.3	4.8	1.5
Traf1	3.5	2.3	9.4	6.1	2.7
Hmox1	2	3.4	7.3	12.3	3.6
Fas	2.7	3	2.6	2.8	−1.1
Bcar3	−2.8	−3.9	3.3	2.3	9.1
Pfkp	−2.2	−2.4	1.4	1.3	3
Gys1	−2.7	−2.4	−1.2	−1.1	2.2
Bnip3	−3.6	−4.4	1.3	1.1	4.9
Ndrg1	−2.3	−3.4	9.1	6.2	21.4
Ogt	−2.1	−2.2	1.5	1.4	3.1
Slc2a1	−2.2	−2.8	5.3	4.1	11.8
Pgm2	−2.3	−2.6	1.3	1.2	3.1
Agpat9	−3.2	−4.7	3.6	2.5	11.5
Camk2d	−2.8	−3.2	3.7	3.2	10.3
Hivep3	−2.4	−2.7	1.5	1.4	3.7

The list represents fold-change between BCG, H37Ra, and *M. smeg* infection compared to un-infected control (Ctl).

Two processes in MTB-induced host cell death have been widely described: anti-apoptosis, which maintains host cell viability, and necrosis, which destroys host cells to disseminate infection [15]. Identification of pathogenic-specific anti-apoptotic genes may pave the way for innovative therapeutic strategies for MTB infection. In fact, carbon monoxide, the principal metabolite from HMOX1 (HO-1), has been recognized as a novel bactericidal molecule in the host defense against microbes such as MTB [17].

### Identification of ITGA5 and ITGAV as novel PRRs with high MOI challenge

Macrophages are the primary targets of MTB infection. Entry of the pathogen through the macrophage plasma membrane occurs either by engulfment through stimulated TLRs, or sinking of the bacilli into the cell through up regulation of integrins such as CD11b (alphaM, Itgam) and CD11c (alphax, Itgax) , as shown in experiments using low MOI conditions [18].

**Figure 5:**
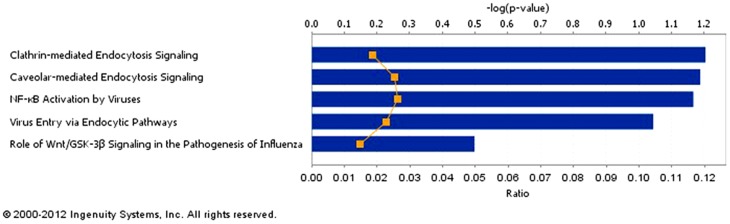
Pathogen-influenced canonical pathways identified from up-regulated cell surface proteins. Up-regulated plasma membrane protein genes were grouped into canonical pathways by Pathway Analysis. Five overrepresented pathogen-influenced canonical pathways were identified, with the up-regulated plasma membrane receptors comprising these pathways listed at in Table 2.

As neither TLRs nor other classical plasma membrane PRRs including CD11b/c were up-regulated by mycobacterial infection in this study, in order to identify other potential phagocytic receptors induced by high mycobacterial MOI, we looked for plasma membrane receptors among our list of up-regulated genes. Within these genes, five cell-surface receptors were identified from pathogen-influenced signaling pathways (Figure 5 and Table 2). Among them, ITGAV was stimulated by all three species, and ITGA5 was up-regulated only by BCG and H37Ra infection.

**Table 2:** Up-regulated plasma membrane receptors identified from the over-represented canonical pathways shown in Figure 5.

Ingenuity Canonical Pathways	−log(p-value)*	Plasma membrane receptors
Clathrin-mediated Endocytosis Signaling	2.58E00	LDLR,ITGA5,TFRC
Caveolar-mediated Endocytosis Signaling	2.15E00	ITGAV,ITGA5
NF-κB Activation by Viruses	2.12E00	ITGAV,ITGA5
Virus Entry via Endocytic Pathways	1.99E00	ITGA5,TFRC
Role of Wnt/GSK-3β Signaling in the Pathogenesis of Influenza	9.57E-01	FZD5

* The P-value was calculated using Fischer's exact test determining the probability of the association between the genes in the canonical pathway.

Integrins are heterodimeric receptors of alpha and beta transmembrane subunits that mediate intercellular communication through cell-extracellular matrix interactions and cell-cell interactions. Several human pathogens are known to bind integrins directly and use integrin-mediated signaling to invade various types of host cells including macrophages [19], [20]. Two integrins, Itgam (CD11b) and Itgax (CD11c), which were not differentially-expressed in our study, have been implicated in mycobacterial entry [21]. Because of cell surface accessibility, integrins have been attractive pharmacological targets. Several integrin-targeted antagonists have been studied in clinical trials for autoimmune diseases such as HIV infection, multiple sclerosis, psoriasis and rheumatoid arthritis [22]. As the mRNA levels of plasma membrane PPRs, including CD11b/c, were not up-regulated in our study, it is reasonable to assume that ITGA5 and ITGAV were instead the possible predominant integrins used by mycobacteria to enter macrophages under high MOI conditions [19], [20].

### Identification of SRC as having a central role in multiple signaling pathways

**Figure 6:**
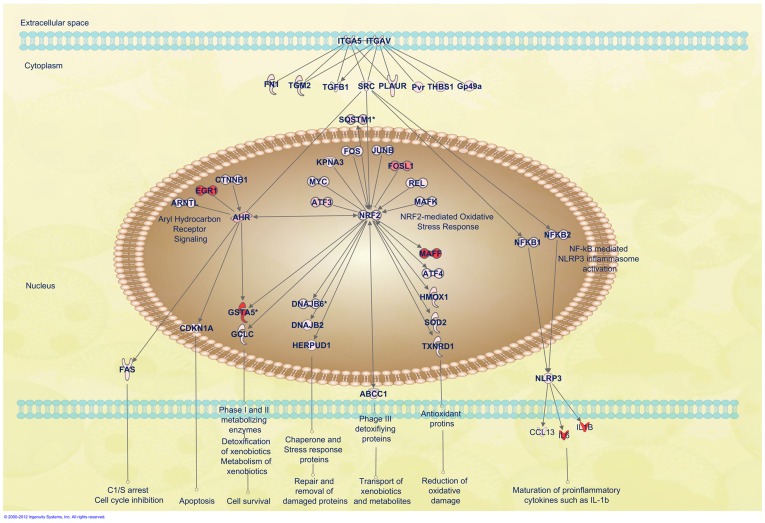
Diagram of integrin (ITGA5 and ITGAV) and SRC signaling pathways. Shown are AhR signaling, the NRF2-mediated oxidative stress response, and the NLRP3 inflammasome activation pathway regulated by ITGA5/ITGAV and SRC axis. Up-regulated genes downstream of ITGA5/ITGAV and SRC were linked to these three identified signaling pathways with data only displayed from BCG infection. For details on gene abbreviations and fold changes compared with control, see Table S3. The network is graphically represented as nodes (gene products) and edges (the biological relationship between nodes). Red-shaded nodes represent up-regulated genes.

To investigate the downstream effects of ITGAV and ITGA5 binding by MTB, we looked for up-regulated genes in the integrin activation pathway. Eight genes with direct connections to ITGAV or ITGA5 were differentially expressed (Figure 6 and Table S4), and 49 additional genes downstream from those eight were differentially expressed. Interestingly, pathway analysis indicates that SRC is a key gene controlling most of the up-regulated genes (29 of 49; Table S5). In addition, SRC is one of the few genes (SRC, Plaur, Tgfb1 and Tgm2) regulated by both ITGA5 and ITGAV (Figure 6).

As a non-receptor tyrosine kinase and the first oncogene discovered, SRC has been a well-studied target in cancer therapy [23]. It has been implicated in many intracellular signaling pathways initiated by a varied set of receptors from integrins to TLRs [24], [25]. SRC tyrosine kinases transmit integrin-dependent signals for cell movement and proliferation [25]. Rapid activation of SRC following integrin-ligand interactions contributes to reinforcement of initial integrin-mediated adhesion by activating downstream kinases, adaptors, and cytokine/chemokine receptors [25]–[28]. Functional studies show that SRC has a key role in host defense and inflammation [24]. In viral disease, SRC kinase inhibitors result in diminution of virus-induced angiogenesis and significantly reduce the accumulation of inflammatory cytokine mRNA. These studies suggest a potential role for SRC in anti-pathogen signaling in macrophages [29], [30]. Several SRC inhibitors have been in clinical use or in late-stages clinical trials for solid tumors [31].

Less attention has been given to the role of SRC in MTB innate immune responses. SRC has been suggested to be the crucial regulator after cellular responses of virulent MTB infection with low MOI (MOI = 10) for 8 hours [27]. In our study, SRC was stimulated by all three Mycobacterium species, including non-pathogenic *M. smeg*. Therefore, we decided to focus on SRC in order to systematically understand its role in high MOI mycobacterial infection. Several distinct signaling pathways downstream of SRC, such as AhR, NRF2-mediated oxidative stress response, and NLRP3 inflammasome activation, are activated in response to MTB infection (Figure 6).

### Stimulation of AhR signaling pathway regulated by SRC

Endogenous AhR activation enhances innate immune responses and plays a cytoprotective role following infection [32], [33]. However, the potential involvement of AhR-mediated immunomodulation has never been investigated in mycobacterial infection [34]. AhR is up-regulated by infection with all mycobacterial species with the highest stimulation by BCG. A fascinating observation is that the genes upstream of AhR are directly involved in apoptosis (Figure 6 and Table S4). Among these genes, cytosolic SRC is likely the best candidate for initializing AhR activation, as other upstream genes, such as ARNT, EGR1, CTNNB1 and NRF2, are located in the nucleus. However, there is a possibility that the cytosolic debris of the mycobacterium, such as its cell wall components, might act as ligands to activate AhR directly [35]. An activated AhR pathway eventually triggers several cellular functions such as apoptosis, cell survival and cell cycle inhibition through the stimulation of FAS, CDKN1A, GSTA5 (GSTA2) and GCLC (Figure 6). Genes downstream of AhR, FAS and GSTA5, are stimulated more predominantly by BCG and H37Ra infection. GSTA5 was considered as one of the possible pathogenic-related genes discussed earlier (Figure 6 and Table S4). AhR has been suggested as a potential therapeutic target for immune-mediated diseases. A recent study indicated that an immunomodulatory drug, leflunomide, is an AhR agonist. The study paves the way for the feasibility of developing more AhR-targeted therapeutics, especially in MTB treatment [36].

### Activation of the NRF2 signaling pathway through a high MOI challenge

As a ubiquitous master transcription regulator of various stress response pathways, NRF2 can be activated by various stimuli, including oxidants, pro-oxidants, and antioxidants. Many genes, including SRC and AhR, directly regulate NRF2 (Figure 6). NRF2 expression is activated by all three mycobacterial species, with highest activation by BCG (Table S4). As the crucial redox- sensitive transcription factor controlling many biological processes, NRF2 (NFE2L2) subsequently activates many target genes of phase I-III enzymes, as well as antioxidant proteins, to promote detoxification and antioxidation, eventually determining the cell's fate in survival/death signaling (Figure 6).

In the clinic, as a late-stage transcription factor, NRF2 was predominantly detected within primary granulomas in MTB-infected lung lesions, and its detection became more prominent as the infection progressed [37]. With few clinical studies and lack of systematic analysis of oxidative stress and antioxidant responses in the host response to mycobacterial infection, these data allow for a global view of host antioxidant defenses. In fact, investigational drugs targeting NRF2 have been evaluated in oxidative stress-related pathological conditions [38].

### Activation of NLRP3 inflammasomes without ESAT-6 as the mandatory stimulator

NLRP3 inflammasomes are multimolecular platforms working together with plasma membrane PRRs to control synthesis, maturation and secretion of proinflammatory cytokines [39]. Recent studies have suggested that only pathogenic Mycobacteria at low MOI induce activation of the NLRP3 inflammasome [39]. We found that NLRP3 was also up-regulated by pathogenic BCG, H37Ra and non-pathogenic *M. smeg* with high MOI conditions (Figure 6 and Table 2). NLRP3 inflammasome activation and assembly requires a combination of signals from various stimulated nuclear factor kappa B (NFkB) proteins and the mandatory second stimulation signal of early secreted antigenic target 6-kDa antigen (ESAT-6) from certain mycobacterial species [40], [41]. The ESAT-6/culture filtrate protein 10 (CFP-10) complex secreted by the ESX-1 secretion system, is encoded in the RD1 region, which is present in all strains of MTB and *M. smeg*, but not in BCG [42]. Our data from activated NLRP3 in BCG suggests that ESAT-6/CFP-10 from the RD1 region may not be the only obligatory second signal for NLRP3 activation. As ITGAV and ITGA5 are the proposed PRRs for bacillary entry in this high mycobacterial MOI study, it is rational to assume that maturation and secretion of proinflammatory cytokines occur through the NFkB-mediated NLRP3 inflammasome, which is activated by the integrin/SRC axis (Figure 6). Preclinical studies demonstrate that inducible NLRP3 inflammasome-mediated innate immune responses effectively reduce the intracellular pathogen load. Targeting the NLRP3 inflammasome opens a door for developing a broad-spectrum anti-infective agent [43].

### Host factors in antimicrobial treatment

Host-directed anti-infective therapy has emerged as a concept to develop broad-spectrum therapies. Recently, a functional screen identified a set of host factors involving MTB survivability [44]–[46]. Many of these genes were differentially expressed in our study (Table S6). A promising host-directed immunomodulatory approach is to enhance host-protective antimicrobial immunity through agonists of cell surface receptors, including PRRs such as TLRs and NLRs [47]. One encouraging clinical trial using this approach demonstrated that autologously transplanted CD34+ hematopoietic stem cells with siRNA-attenuated CCR5 receptors later develop into HIV-1 resistant T-cells and macrophages to further protect against HIV-1 infection [48], [49]. Our systematic analysis greatly enhances insights into the complex network of host-pathogen interactions. With a shortlist of potential host targets, sound identification of drug targets for host-directed anti-mycobacterial therapies could be initialized.

In summary, our genome-wide expression profiling studies provide a better understanding of the interplay between a high intracellular load of mycobacteria and macrophage host cells by examining infection responses among Mycobacterial species. The integrated results are a valuable insight into high-load mycobacterial infections that mimic clinical disseminated infection [50], [51] and the formation of granulomas within host tissues [52], [53]. This has allowed us to identify clusters of coordinately regulated genes that may facilitate the discovery of new therapeutic and diagnostic targets for MTB infection.

## Supporting Information

Table S1 **Biological process enrichment classification of altered genes.** Up-regulated (A) and down-regulated genes (B) were classified according to PANTHER pathway analysis software with the cut-off value p<0.01. Symbols used in the table: #, number of genes; expected, the number of genes expected in the list for this PANTHER category, based on the reference list; +/−, over representation of a category is denoted by a ‘+’ sign and under representation by a ‘−’ sign.
(DOCX)

Table S2 **qPCR and array fold-change comparison.** Log2 fold-change from the results of qPCR and fold-change from microarray experiments are listed for validation of differential expression from a selected set of 31 genes by quantitative real time PCR (qPCR) (figure 3) with respect to mycobacterial infection samples comparing to uninfected control.
(DOCX)

Table S3 **List of 2 fold-changes between the infection and control groups.** The list represents the fold-change between BCG, H37Ra, M. smeg infection and un-infected control. Red highlight indicates fold-change (FC) of BCG or H37Ra compared to M.smeg ≥2, and green indicates ≤−2 fold.
(DOCX)

Table S4 **List of genes downstream from the ITGA5/ITGAV and SRC axis from** **figure 6** **.** The list represents genes differentially expressed between BCG, H37Ra and M. smeg infection compared to un-infected control (Ctl). The genes were classified as groups directly downstream of ITGA5/ITGAV, or from AHR signaling, NF-kB mediated NLRP3 inflammasome activation and NRF2-mediated oxidative stress response pathways.
(DOCX)

Table S5 **List of genes downstream from eight genes regulated by ITGAV and/or ITGA5.** The list represents the eight genes (FN1, Gp49a, PLAUR, SRC, TGFB1, TGM2, THBS1 and Pvr) defined by pathway analysis. The “Gene” column indicates genes directly downstream from these eight genes with exception of Pvr which had no direct downstream interactions defined by Ingenuity Pathway Analysis.
(DOCX)

Table S6 **List of regulated genes identified by two previous functional screens.** The list represents the list of the host factors identified from two previous siRNA screens [44]–[46] and also regulated in our study. The list demonstrated the fold-change of the regulated genes from BCG, H37Ra and M. smeg compared to un-infected control.
(DOCX)

Table S7 **List of the primers used for qPCR.** The list represents the list of primer sequences used for SYBR Green quantitative real time PCR (qPCR) (figure 3) with additional primers for 3 housekeeping genes.
(DOCX)
