# Supplementary material for: Transcriptome Analysis Reveals Novel Entry Mechanisms and a Central Role of SRC in Host Defense during High Multiplicity Mycobacterial Infection
Source: PLoS One. 2013 Jun 18;8(6):e65128. doi: 10.1371/journal.pone.0065128 (PMC3688827; doi:10.1371/journal.pone.0065128)
Supplement: Table S1 — Biological process enrichment classification of altered genes. Up-regulated (A) and down-regulated genes (B) were classified according to PANTHER pathway analysis software with the cut-off value p<0.01. Symbols used in the table: #, number of genes; expected, the number of genes expected in the list for this PANTHER category, based on the reference list; +/−, over representation of a category is denoted by a ‘+’ sign and under representation by a ‘−’ sign. (DOCX) [file pone.0065128.s001.docx]

| **Table A** |  |  |  |  |  |
| --- | --- | --- | --- | --- | --- |
| **Biological Process** | **Mus musculus #** | **UP regulated #** | **Expected** | **Over/under** | **P-value** |
| Unclassified | 11454 | 166 | 282.58 | - | 2.48E-19 |
| cytokine-mediated signaling pathway | 282 | 38 | 6.96 | + | 1.84E-14 |
| intracellular signaling cascade | 1083 | 74 | 26.72 | + | 1.22E-12 |
| immune system process | 1898 | 104 | 46.82 | + | 4.47E-12 |
| cell communication | 4194 | 179 | 103.47 | + | 7.36E-12 |
| signal transduction | 4013 | 173 | 99 | + | 9.91E-12 |
| metabolic process | 8626 | 292 | 212.81 | + | 1.09E-08 |
| cellular process | 6077 | 220 | 149.92 | + | 4.58E-08 |
| primary metabolic process | 8197 | 276 | 202.23 | + | 1.18E-07 |
| MAPKKK cascade | 278 | 27 | 6.86 | + | 6.05E-07 |
| cell-cell signaling | 853 | 49 | 21.04 | + | 1.27E-05 |
| protein amino acid phosphorylation | 700 | 43 | 17.27 | + | 1.38E-05 |
| JNK cascade | 97 | 14 | 2.39 | + | 3.90E-05 |
| response to stimulus | 1568 | 72 | 38.68 | + | 7.32E-05 |
| mesoderm development | 819 | 45 | 20.21 | + | 1.44E-04 |
| JAK-STAT cascade | 94 | 13 | 2.32 | + | 1.67E-04 |
| protein modification process | 1253 | 60 | 30.91 | + | 1.97E-04 |
| I-kappaB kinase/NF-kappaB cascade | 83 | 12 | 2.05 | + | 2.76E-04 |
| negative regulation of apoptosis | 170 | 17 | 4.19 | + | 3.20E-04 |
| apoptosis | 715 | 40 | 17.64 | + | 3.86E-04 |
| immune response | 562 | 34 | 13.86 | + | 4.40E-04 |
| hemopoiesis | 103 | 13 | 2.54 | + | 4.49E-04 |
| regulation of transcription from RNA polymerase II promoter | 1588 | 68 | 39.18 | + | 1.59E-03 |
| transmembrane receptor protein serine/threonine kinase signaling pathway | 43 | 8 | 1.06 | + | 2.58E-03 |
| **Table B** |  |  |  |  |  |
| **Biological Process** | **Mus musculus #** | **Down-regulated #** | **Expected** | **Over/under** | **P value** |
| cell cycle | 1256 | 154 | 53.53 | + | 5.80E-29 |
| DNA metabolic process | 384 | 78 | 16.37 | + | 1.30E-26 |
| Unclassified | 11454 | 315 | 488.17 | - | 1.40E-24 |
| primary metabolic process | 8197 | 516 | 349.35 | + | 2.50E-23 |
| metabolic process | 8626 | 531 | 367.64 | + | 5.40E-22 |
| cellular process | 6077 | 408 | 259 | + | 1.40E-21 |
| nucleobase, nucleoside, nucleotide and nucleic acid metabolic process | 3636 | 268 | 154.97 | + | 2.10E-17 |
| DNA replication | 173 | 40 | 7.37 | + | 5.10E-15 |
| protein amino acid phosphorylation | 700 | 85 | 29.83 | + | 5.60E-15 |
| DNA repair | 185 | 40 | 7.88 | + | 4.60E-14 |
| protein modification process | 1253 | 115 | 53.4 | + | 5.30E-12 |
| establishment or maintenance of chromatin architecture | 268 | 40 | 11.42 | + | 4.50E-09 |
| mitosis | 392 | 49 | 16.71 | + | 1.20E-08 |
| organelle organization | 298 | 40 | 12.7 | + | 9.40E-08 |
| intracellular signaling cascade | 1083 | 91 | 46.16 | + | 2.40E-07 |
| DNA recombination | 77 | 19 | 3.28 | + | 3.60E-07 |
| transcription from RNA polymerase II promoter | 2101 | 143 | 89.54 | + | 5.00E-06 |
| chromosome segregation | 125 | 21 | 5.33 | + | 3.50E-05 |
| protein metabolic process | 3454 | 207 | 147.21 | + | 4.90E-05 |
| transcription | 2196 | 143 | 93.59 | + | 6.30E-05 |
| regulation of transcription from RNA polymerase II promoter | 1588 | 108 | 67.68 | + | 3.00E-04 |
| protein transport | 1259 | 89 | 53.66 | + | 5.90E-04 |
| intracellular protein transport | 1259 | 89 | 53.66 | + | 5.90E-04 |
| apoptosis | 715 | 57 | 30.47 | + | 1.40E-03 |
| response to pheromone | 273 | 0 | 11.64 | - | 1.40E-03 |
| cellular component organization | 1056 | 74 | 45.01 | + | 5.30E-03 |
| phosphate metabolic process | 136 | 18 | 5.8 | + | 5.90E-03 |
| response to stress | 278 | 28 | 11.85 | + | 6.90E-03 |
| MAPKKK cascade | 278 | 28 | 11.85 | + | 6.90E-03 |
