# Supplementary material for: Transcriptome Analysis Reveals Novel Entry Mechanisms and a Central Role of SRC in Host Defense during High Multiplicity Mycobacterial Infection
Source: PLoS One. 2013 Jun 18;8(6):e65128. doi: 10.1371/journal.pone.0065128 (PMC3688827; doi:10.1371/journal.pone.0065128)
Supplement: Table S2 — qPCR and array fold-change comparison. Log2 fold-change from the results of qPCR and fold-change from microarray experiments are listed for validation of differential expression from a selected set of 31 genes by quantitative real time PCR (qPCR) (figure 3) with respect to mycobacterial infection samples comparing to uninfected control. (DOCX) [file pone.0065128.s002.docx]

| **Sample Name** | **Gene Symbol** | **qPCR Log2 Fold Change** | **Array Log2 Fold Change** |
| --- | --- | --- | --- |
| BCG | AHR | 4.4 | 3.7 |
| H37Ra |  | 2.9 | 3.3 |
| M.Smeg |  | 3.1 | 2.8 |
| BCG | ATF3 | 4.4 | 3.4 |
| H37Ra |  | 3.9 | 3.2 |
| M.Smeg |  | 4.8 | 3.4 |
| BCG | BCL2A1A | 5.1 | 3.8 |
| H37Ra |  | 2.5 | 2.8 |
| M.Smeg |  | 5.1 | 3.5 |
| BCG | CCL3 | 5.9 | 3.8 |
| H37Ra |  | 3.8 | 3.6 |
| M.Smeg |  | 6.2 | 3.8 |
| BCG | CCL4 | 5.3 | 3.1 |
| H37Ra |  | 4.2 | 2.9 |
| M.Smeg |  | 5.1 | 3.0 |
| BCG | CCL5 | 4.5 | 2.6 |
| H37Ra |  | 2.3 | 1.5 |
| M.Smeg |  | 4.4 | 2.0 |
| BCG | CCL7 | 4.8 | 4.2 |
| H37Ra |  | 3.8 | 3.9 |
| M.Smeg |  | 4.9 | 3.7 |
| BCG | CSF1 | 3.4 | 2.3 |
| H37Ra |  | 4.2 | 2.2 |
| M.Smeg |  | 4.9 | 2.6 |
| BCG | CXCL10 | 4.3 | 2.3 |
| H37Ra |  | 2.1 | 1.7 |
| M.Smeg |  | 3.1 | 0.9 |
| BCG | CXCL2 | 6.4 | 4.7 |
| H37Ra |  | 6.2 | 4.6 |
| M.Smeg |  | 6.6 | 4.7 |
| BCG | ICAM1 | 1.2 | 1.7 |
| H37Ra |  | 1.4 | 1.5 |
| M.Smeg |  | 1.8 | 0.5 |
| BCG | IL10 | 5.9 | 4.8 |
| H37Ra |  | 4.4 | 3.2 |
| M.Smeg |  | 5.7 | 3.9 |
| BCG | IL1A | 6.9 | 6.7 |
| H37Ra |  | 6.0 | 5.4 |
| M.Smeg |  | 7.0 | 6.7 |
| BCG | IL1B | 6.6 | 5.2 |
| H37Ra |  | 5.4 | 3.6 |
| M.Smeg |  | 6.5 | 4.9 |
| BCG | IL1RN | 5.8 | 4.2 |
| H37Ra |  | 5.2 | 3.5 |
| M.Smeg |  | 6.2 | 4.5 |
| BCG | IL6 | 7.5 | 8.8 |
| H37Ra |  | 7.0 | 7.9 |
| M.Smeg |  | 7.5 | 8.4 |
| BCG | IRG1 | 5.5 | 5.0 |
| H37Ra |  | 5.1 | 5.0 |
| M.Smeg |  | 5.8 | 5.2 |
| BCG | ITGA5 | 1.9 | 1.2 |
| H37Ra |  | 2.0 | 1.1 |
| M.Smeg |  | 3.4 | 0.5 |
| BCG | ITGAV | 2.4 | 1.7 |
| H37Ra |  | 1.9 | 1.7 |
| M.Smeg |  | 3.0 | 1.6 |
| BCG | JUNB | 2.9 | 2.6 |
| H37Ra |  | 2.9 | 2.3 |
| M.Smeg |  | 3.7 | 2.5 |
| BCG | NLRP3 | 2.7 | 1.9 |
| H37Ra |  | 2.4 | 1.4 |
| M.Smeg |  | 3.2 | 1.4 |
| BCG | OSM | 5.4 | 4.1 |
| H37Ra |  | 4.9 | 3.7 |
| M.Smeg |  | 5.5 | 3.8 |
| BCG | TNF | 5.3 | 4.1 |
| H37Ra |  | 3.9 | 3.6 |
| M.Smeg |  | 5.4 | 4.1 |
| BCG | TNFAIP3 | 5.0 | 4.1 |
| H37Ra |  | 4.6 | 3.9 |
| M.Smeg |  | 5.4 | 4.6 |
| BCG | TNFRSF1B | 4.1 | 2.8 |
| H37Ra |  | 3.5 | 2.7 |
| M.Smeg |  | 4.6 | 3.2 |
| BCG | AURKB | -1.1 | -2.5 |
| H37Ra |  | -1.4 | -2.2 |
| M.Smeg |  | -0.9 | -1.9 |
| BCG | CCND3 | -2.0 | -2.4 |
| H37Ra |  | -2.1 | -2.3 |
| M.Smeg |  | -0.7 | -2.1 |
| BCG | CENPF | -0.9 | -2.4 |
| H37Ra |  | -1.7 | -2.7 |
| M.Smeg |  | -0.4 | -2.0 |
| BCG | IL6RA | -0.6 | -1.7 |
| H37Ra |  | -1.0 | -1.8 |
| M.Smeg |  | -0.2 | -2.6 |
| BCG | KIF11 | -1.4 | -2.6 |
| H37Ra |  | -1.7 | -2.4 |
| M.Smeg |  | -0.2 | -2.2 |
| BCG | MAF | -0.2 | -1.6 |
| H37Ra |  | -2.1 | -1.3 |
| M.Smeg |  | -2.0 | -1.8 |
