# Supplementary material for: Transcriptome Analysis Reveals Novel Entry Mechanisms and a Central Role of SRC in Host Defense during High Multiplicity Mycobacterial Infection
Source: PLoS One. 2013 Jun 18;8(6):e65128. doi: 10.1371/journal.pone.0065128 (PMC3688827; doi:10.1371/journal.pone.0065128)
Supplement: Table S3 — List of 2 fold-changes between the infection and control groups. The list represents the fold-change between BCG, H37Ra, M. smeg infection and un-infected control. Red highlight indicates fold-change (FC) of BCG or H37Ra compared to M.smeg ≥2, and green indicates ≤−2 fold. (DOCX) [file pone.0065128.s003.docx]

| **Affymetrix Probe Set ID** | **Gene Symbol** | **BCG/ M.smeg** | **H37Ra/M.smeg** | **BCG/Ctl** | **H37Ra/Ctl** | **M.smeg/Ctl** |
| --- | --- | --- | --- | --- | --- | --- |
| 1416926_at | Trp53inp1 | 14.8 | 21.1 | 1.5 | 2.1 | -9.9 |
| 1445678_at | --- | 16.0 | 17.5 | 21.8 | 23.9 | 1.4 |
| 1449002_at | Phlda3 | 4.8 | 7.9 | 1.4 | 2.4 | -3.3 |
| 1417487_at | Fosl1 | 2.8 | 3.9 | 23.1 | 32.3 | 8.3 |
| 1442532_at | --- | 5.1 | 3.4 | 13.7 | 9.3 | 2.7 |
| 1448239_at | Hmox1 | 2.0 | 3.4 | 7.3 | 12.3 | 3.6 |
| 1417902_at | Slc19a2 | 2.5 | 3.4 | 1.6 | 2.1 | -1.6 |
| 1453675_at | Slc16a10 | 4.7 | 3.4 | 2.9 | 2.1 | -1.6 |
| 1429350_at | Eid3 | 5.1 | 3.1 | 19.2 | 11.8 | 3.8 |
| 1458708_at | --- | 3.3 | 3.1 | 2.1 | 2.0 | -1.6 |
| 1442878_at | Prdx6 | 2.8 | 3.1 | 4.3 | 4.8 | 1.5 |
| 1435484_at | Slc5a3 | 3.9 | 3.1 | 2.7 | 2.1 | -1.5 |
| 1460251_at | Fas | 2.7 | 3.0 | 2.6 | 2.8 | -1.1 |
| 1422134_at | Fosb | 4.7 | 2.9 | 6.5 | 4.1 | 1.4 |
| 1454742_at | Rasgef1b | 2.5 | 2.9 | 2.5 | 2.9 | -1.0 |
| 1438900_at | Sacm1l | 3.4 | 2.7 | 4.9 | 4.0 | 1.4 |
| 1421041_s_at | Gsta1 | 2.5 | 2.7 | 9.2 | 9.9 | 3.7 |
| 1432820_at | Psmd7 | 2.9 | 2.6 | 2.2 | 1.9 | -1.3 |
| 1434152_at | Apol7c | 6.0 | 2.6 | 4.1 | 1.8 | -1.5 |
| 1439098_at | E230013L22Rik | 3.2 | 2.5 | 6.8 | 5.4 | 2.2 |
| 1457458_at | Zc3h4 | 3.5 | 2.5 | 2.5 | 1.8 | -1.4 |
| 1431940_at | LOC74457 | 2.2 | 2.5 | 5.9 | 6.6 | 2.6 |
| 1435584_at | A630033H20Rik | 2.5 | 2.5 | 3.2 | 3.2 | 1.3 |
| 1419203_at | Gm16516 | 2.4 | 2.4 | 2.9 | 3.0 | 1.2 |
| 1457035_at | AI607873 | 2.6 | 2.4 | 3.4 | 3.1 | 1.3 |
| 1433451_at | Cdk5r1 | 2.7 | 2.3 | 3.6 | 3.2 | 1.4 |
| 1436524_at | 4833438C02Rik | 2.7 | 2.3 | 2.7 | 2.4 | 1.0 |
| 1435684_at | Abcc5 | 3.3 | 2.3 | 3.3 | 2.3 | 1.0 |
| 1445452_at | Traf1 | 3.5 | 2.3 | 9.4 | 6.1 | 2.7 |
| 1431347_at | 5730407M17Rik | 2.3 | 2.3 | 2.5 | 2.5 | 1.1 |
| 1416259_at | Pex12 | 2.3 | 2.3 | 2.7 | 2.7 | 1.2 |
| 1436329_at | Egr3 | 2.5 | 2.2 | 7.4 | 6.7 | 3.0 |
| 1423100_at | Fos | 2.4 | 2.2 | 2.2 | 1.9 | -1.1 |
| 1418203_at | Pmaip1 | 2.0 | 2.1 | 3.9 | 4.2 | 1.9 |
| 1446304_at | --- | 2.2 | 2.1 | 2.6 | 2.5 | 1.2 |
| 1421040_a_at | Gsta2 | 2.2 | 2.1 | 181.3 | 173.4 | 83.9 |
| 1435774_at | LOC106740 | -2.3 | -2.2 | 1.1 | 1.2 | 2.5 |
| 1434951_at | Armc8 | -2.3 | -2.2 | 1.2 | 1.3 | 2.7 |
| 1439797_at | Ppard | -2.0 | -2.2 | 2.0 | 1.9 | 4.1 |
| 1425516_at | Ogt | -2.1 | -2.2 | 1.5 | 1.4 | 3.1 |
| 1416776_at | Crym | -2.0 | -2.3 | 2.1 | 1.8 | 4.2 |
| 1423810_at | Ppme1 | -2.0 | -2.3 | 1.3 | 1.1 | 2.7 |
| 1416069_at | Pfkp | -2.2 | -2.4 | 1.4 | 1.3 | 3.0 |
| 1416737_at | Gys1 | -2.7 | -2.4 | -1.2 | -1.1 | 2.2 |
| 1426810_at | Kdm3a | -2.0 | -2.5 | 1.3 | 1.1 | 2.7 |
| 1427143_at | Kdm5b | -2.2 | -2.5 | 1.9 | 1.7 | 4.3 |
| 1451149_at | Pgm2 | -2.3 | -2.6 | 1.3 | 1.2 | 3.1 |
| 1458802_at | Hivep3 | -2.4 | -2.7 | 1.5 | 1.4 | 3.7 |
| 1460013_at | Nlgn2 | -4.8 | -2.8 | 4.0 | 7.0 | 19.4 |
| 1426600_at | Slc2a1 | -2.2 | -2.8 | 5.3 | 4.1 | 11.8 |
| 1424011_at | Aqp9 | -2.3 | -2.9 | 1.7 | 1.4 | 4.0 |
| 1445443_at | --- | -2.5 | -2.9 | 1.5 | 1.3 | 3.7 |
| 1417149_at | P4ha2 | -2.9 | -3.0 | 1.5 | 1.5 | 4.4 |
| 1449954_at | Hyal1 | -3.7 | -3.0 | 1.5 | 1.8 | 5.4 |
| 1450376_at | Mxi1 | -3.0 | -3.2 | -1.0 | -1.1 | 2.9 |
| 1456844_at | Camk2d | -2.8 | -3.2 | 3.7 | 3.2 | 10.3 |
| 1418829_a_at | Eno2 | -3.1 | -3.3 | 4.7 | 4.4 | 14.8 |
| 1423413_at | Ndrg1 | -2.3 | -3.4 | 9.1 | 6.2 | 21.4 |
| 1427747_a_at | Lcn2 | -3.0 | -3.5 | 9.6 | 8.2 | 28.3 |
| 1415936_at | Bcar3 | -2.8 | -3.9 | 3.3 | 2.3 | 9.1 |
| 1417408_at | F3 | -2.1 | -4.2 | 50.8 | 25.6 | 107.4 |
| 1422612_at | Hk2 | -3.1 | -4.4 | 2.3 | 1.6 | 7.3 |
| 1422470_at | Bnip3 | -3.6 | -4.4 | 1.3 | 1.1 | 4.9 |
| 1454799_at | Agpat9 | -3.2 | -4.7 | 3.6 | 2.5 | 11.5 |
| 1426743_at | Appl2 | -3.6 | -5.0 | 1.2 | -1.2 | 4.3 |
| 1452163_at | Ets1 | -3.9 | -5.2 | 4.5 | 3.4 | 17.6 |
| 1421207_at | Lif | -2.3 | -8.0 | 20.6 | 5.9 | 47.6 |
| 1419427_at | Csf3 | -3.0 | -9.2 | 53.8 | 17.4 | 160.8 |
| 1436590_at | Ppp1r3b | -9.9 | -13.0 | 1.8 | 1.3 | 17.5 |
| 1436538_at | Ankrd37 | -6.9 | -13.9 | 5.6 | 2.8 | 38.2 |
