# Supplementary material for: Transcriptome Analysis Reveals Novel Entry Mechanisms and a Central Role of SRC in Host Defense during High Multiplicity Mycobacterial Infection
Source: PLoS One. 2013 Jun 18;8(6):e65128. doi: 10.1371/journal.pone.0065128 (PMC3688827; doi:10.1371/journal.pone.0065128)
Supplement: Table S4 — List of genes downstream from the ITGA5/ITGAV and SRC axis from figure 6 . The list represents genes differentially expressed between BCG, H37Ra and M. smeg infection compared to un-infected control (Ctl). The genes were classified as groups directly downstream of ITGA5/ITGAV, or from AHR signaling, NF-kB mediated NLRP3 inflammasome activation and NRF2-mediated oxidative stress response pathways. (DOCX) [file pone.0065128.s004.docx]

| **Affymetrix Probeset ID** | **Gene Symbol** | **BCG/ctl** | **H37Ra/ctl** | **M.smeg/ctl** | **Group** |
| --- | --- | --- | --- | --- | --- |
| 1422631_at | AHR | 12.7 | 9.8 | 6.8 | AHR signaling |
| 1425099_a_at | ARNTL | 1.3 | 1.2 | 2.1 | AHR signaling |
| 1424638_at | CDKN1A | 3 | 3 | 1.9 | AHR signaling |
| 1450008_a_at | CTNNB1 | 2.4 | 2.1 | 2.5 | AHR signaling |
| 1417065_at | EGR1 | 36.2 | 20.8 | 13 | AHR signaling |
| 1460251_at | FAS | 2.6 | 2.8 | -1.1 | AHR signaling |
| 1421040_a_at | GSTA2 | 181.3 | 173.4 | 83.9 | AHR signaling |
| 1426642_at | FN1 | 2 | 1.7 | 1.6 | Downstream Genes of ITGA5 and ITGAV |
| 1420394_s_at | GP49A | 2.5 | 2.4 | 2.7 | Downstream Genes of ITGA5 and ITGAV |
| 1452521_a_at | PLAUR | 3.7 | 3 | 4.1 | Downstream Genes of ITGA5 and ITGAV |
| 1423905_at | PVR | 2.4 | 2.4 | 2.7 | Downstream Genes of ITGA5 and ITGAV |
| 1450918_s_at | SRC | 7.1 | 5.9 | 6 | Downstream Genes of ITGA5 and ITGAV |
| 1445360_at | TGFB1 | 2.5 | 1.6 | 2 | Downstream Genes of ITGA5 and ITGAV |
| 1417500_a_at | TGM2 | 2.3 | 2 | 2 | Downstream Genes of ITGA5 and ITGAV |
| 1421811_at | THBS1 | 3 | 1.9 | 2.1 | Downstream Genes of ITGA5 and ITGAV |
| 1427705_a_at | NFKB1 | 2.5 | 2.1 | 3.3 | NF-kB mediated NLRP3 inflammasome activation |
| 1425902_a_at | NFKB2 | 3.9 | 2.8 | 2.5 | NF-kB mediated NLRP3 inflammasome activation |
| 1425412_at | NLRP3 | 3.8 | 2.7 | 2.7 | NF-kB mediated NLRP3 inflammasome activation |
| 1444021_at | AI845619 | 7.8 | 8.4 | 4.9 | NRF2-mediated oxidative stress response pathway |
| 1421378_s_at | ABCC1 | 1.6 | 2.1 | 2.7 | NRF2-mediated oxidative stress response pathway |
| 1449363_at | ATF3 | 10.7 | 9.2 | 10.4 | NRF2-mediated oxidative stress response pathway |
| 1448135_at | ATF4 | 2.9 | 2.7 | 3 | NRF2-mediated oxidative stress response pathway |
| 1448657_a_at | DNAJB2 | 2.6 | 2.3 | 1.8 | NRF2-mediated oxidative stress response pathway |
| 1429777_at | DNAJB6 | 3.2 | 2.1 | 2.1 | NRF2-mediated oxidative stress response pathway |
| 1423100_at | FOS | 2.2 | 1.9 | -1.1 | NRF2-mediated oxidative stress response pathway |
| 1417487_at | FOSL1 | 23.1 | 32.3 | 8.3 | NRF2-mediated oxidative stress response pathway |
| 1424296_at | GCLC | 1.6 | 2.1 | 1.4 | NRF2-mediated oxidative stress response pathway |
| 1448185_at | HERPUD1 | 3.1 | 2.2 | 4.8 | NRF2-mediated oxidative stress response pathway |
| 1448239_at | HMOX1 | 7.3 | 12.3 | 3.6 | NRF2-mediated oxidative stress response pathway |
| 1457561_at | ITGA5 | 2.3 | 2.2 | 1.4 | NRF2-mediated oxidative stress response pathway |
| 1421198_at | ITGAV | 3.3 | 3.3 | 2.9 | NRF2-mediated oxidative stress response pathway |
| 1415899_at | JUNB | 6.2 | 5 | 5.6 | NRF2-mediated oxidative stress response pathway |
| 1450385_at | KPNA3 | 2.5 | 2.2 | 2.8 | NRF2-mediated oxidative stress response pathway |
| 1418936_at | MAFF | 37.3 | 25.7 | 23.1 | NRF2-mediated oxidative stress response pathway |
| 1418616_at | MAFK | 4.6 | 4.2 | 2.5 | NRF2-mediated oxidative stress response pathway |
| 1424942_a_at | MYC | 1.7 | 1.6 | 2.6 | NRF2-mediated oxidative stress response pathway |
| 1457117_at | NRF2 | 6 | 3.7 | 2.2 | NRF2-mediated oxidative stress response pathway |
| 1420710_at | REL | 5.8 | 4.4 | 4.4 | NRF2-mediated oxidative stress response pathway |
| 1454976_at | SOD2 | 2.2 | 2 | 2.2 | NRF2-mediated oxidative stress response pathway |
| 1421529_a_at | TXNRD1 | 2.6 | 2.4 | 2.2 | NRF2-mediated oxidative stress response pathway |
