# Supplementary material for: Transcriptome Analysis Reveals Novel Entry Mechanisms and a Central Role of SRC in Host Defense during High Multiplicity Mycobacterial Infection
Source: PLoS One. 2013 Jun 18;8(6):e65128. doi: 10.1371/journal.pone.0065128 (PMC3688827; doi:10.1371/journal.pone.0065128)
Supplement: Table S5 — List of genes downstream from eight genes regulated by ITGAV and/or ITGA5. The list represents the eight genes (FN1, Gp49a, PLAUR, SRC, TGFB1, TGM2, THBS1 and Pvr) defined by pathway analysis. The “Gene” column indicates genes directly downstream from these eight genes with exception of Pvr which had no direct downstream interactions defined by Ingenuity Pathway Analysis. (DOCX) [file pone.0065128.s005.docx]

| **Affymetrix** | **Gene** | **Up Stream Genes** | **BCG/Clt** | **H37Ra/Ctl** | **M.smeg/Ctl** |
| --- | --- | --- | --- | --- | --- |
| 1452595_at | Adamts4 | FN1 | 3.71 | 1.81 | 3.63 |
| 1417164_at | Dusp10 | FN1 | 5.11 | 3.32 | 5.39 |
| 1415806_at | Plat | FN1 | 4.98 | 3.11 | 1.63 |
| 1417654_at | Sdc4 | FN1 | 2.52 | 1.92 | 2.36 |
| 1422054_a_at | Skil | FN1 | 3.51 | 2.90 | 2.35 |
| 1419607_at | Tnf | FN1 | 17.73 | 12.15 | 17.09 |
| 1460469_at | Tnfrsf9 | FN1 | 4.80 | 3.76 | 6.14 |
| 1426065_a_at | Trib3 | FN1 | 4.73 | 3.60 | 8.33 |
| 1421196_at | Ptpn11 | Gp49a | 3.30 | 1.72 | 1.56 |
| 1429777_at | Dnajb6 | PLAUR | 3.18 | 2.10 | 2.05 |
| 1419149_at | Serpine1 | PLAUR | 8.80 | 4.13 | 16.77 |
| 1422716_a_at | Acp1 | SRC | 2.52 | 3.67 | 1.42 |
| 1422631_at | Ahr | SRC | 12.69 | 9.78 | 6.82 |
| 1450457_at | Cbl | SRC | 2.44 | 1.40 | 2.78 |
| 1427095_at | Cdcp1 | SRC | 2.13 | 4.92 | 1.36 |
| 1425855_a_at | Crk | SRC | 2.39 | 1.76 | 2.32 |
| 1460220_a_at | Csf1 | SRC | 4.88 | 4.72 | 6.17 |
| 1450008_a_at | Ctnnb1 | SRC | 2.38 | 2.12 | 2.48 |
| 1446614_at | Dgkz | SRC | 2.54 | 1.96 | 2.86 |
| 1444010_at | Eif4e | SRC | 3.41 | 2.81 | 2.42 |
| 1425733_a_at | Eps8 | SRC | 2.03 | 1.55 | 1.77 |
| 1416129_at | Errfi1 | SRC | 5.64 | 2.79 | 3.92 |
| 1452163_at | Ets1 | SRC | 4.51 | 3.38 | 17.57 |
| 1416268_at | Ets2 | SRC | 2.16 | 2.00 | 2.39 |
| 1460251_at | Fas | SRC | 2.55 | 2.83 | -1.07 |
| 1441894_s_at | Grasp | SRC | 2.05 | 2.02 | 1.10 |
| 1425396_a_at | Lck | SRC | 2.76 | 2.48 | 2.51 |
| 1456772_at | Ncf1 | SRC | 5.10 | 4.66 | 5.21 |
| 1457117_at | Nfe2l2 | SRC | 5.97 | 3.70 | 2.23 |
| 1429887_at | Nos1 | SRC | 2.26 | 1.80 | 1.68 |
| 1416505_at | Nr4a1 | SRC | 16.14 | 19.28 | 8.55 |
| 1419710_at | Nxph3 | SRC | 2.27 | 1.60 | 1.91 |
| 1429527_a_at | Plscr1 | SRC | 2.13 | 1.64 | 2.77 |
| 1439797_at | Ppard | SRC | 2.04 | 1.89 | 4.11 |
| 1453068_at | Prdm2 | SRC | 2.47 | 1.98 | 2.45 |
| 1421196_at | Ptpn11 | SRC | 3.30 | 1.72 | 1.56 |
| 1422240_s_at | Sprr2h | SRC | 763.12 | 185.48 | 395.01 |
| 1450918_s_at | Src | SRC | 7.06 | 5.92 | 6.05 |
| 1460700_at | Stat3 | SRC | 2.25 | 1.68 | 2.93 |
| 1445452_at | Traf1 | SRC | 9.43 | 6.10 | 2.67 |
| 1435825_at | Acvrl1 | TGFB1 | 2.42 | 2.99 | 2.58 |
| 1417065_at | Egr1 | TGFB1 | 36.24 | 20.75 | 13.03 |
| 1417654_at | Sdc4 | TGFB1 | 2.52 | 1.92 | 2.36 |
| 1448135_at | Atf4 | TGM2 | 2.92 | 2.72 | 2.96 |
| 1417654_at | Sdc4 | TGM2 | 2.52 | 1.92 | 2.36 |
| 1417406_at | Sertad1 | TGM2 | 3.00 | 2.95 | 2.93 |
| 1444021_at | AI845619 | TGM2 | 7.75 | 8.37 | 4.90 |
| 1417523_at | Plek | THBS1 | 2.46 | 1.86 | 3.25 |
| 1421811_at | Thbs1 | THBS1 | 2.99 | 1.91 | 2.13 |
