# Supplementary material for: Transcriptome Analysis Reveals Novel Entry Mechanisms and a Central Role of SRC in Host Defense during High Multiplicity Mycobacterial Infection
Source: PLoS One. 2013 Jun 18;8(6):e65128. doi: 10.1371/journal.pone.0065128 (PMC3688827; doi:10.1371/journal.pone.0065128)
Supplement: Table S6 — List of regulated genes identified by two previous functional screens. The list represents the list of the host factors identified from two previous siRNA screens [44]–[46] and also regulated in our study. The list demonstrated the fold-change of the regulated genes from BCG, H37Ra and M. smeg compared to un-infected control. (DOCX) [file pone.0065128.s006.docx]

| **Probe ID** | **Gene** | **BCG/Clt** | **H37Ra/Ctl** | **M.smeg/Ctl** | **Group** |
| --- | --- | --- | --- | --- | --- |
| 1419721_at | HCAR2 | 31.5 | 16.7 | 46.1 | autophagy-regulatory host factors |
| 1448272_at | BTG2 | 2.5 | 2.4 | 2.0 | autophagy-regulatory host factors |
| 1427218_at | KLHL11 | 2.4 | 2.0 | 2.2 | autophagy-regulatory host factors |
| 1416816_at | NEK7 | 2.2 | 1.9 | 2.8 | autophagy-regulatory host factors |
| 1452057_at | ACTR1B | 1.4 | 1.2 | 2.2 | autophagy-regulatory host factors |
| 1451149_at | PGM1 | 1.3 | 1.2 | 3.1 | autophagy-regulatory host factors |
| 1418264_at | CENPK | -1.5 | -1.4 | -2.7 | autophagy-regulatory host factors |
| 1450685_at | ARPP19 | -2.1 | -1.5 | -2.1 | autophagy-regulatory host factors |
| 1451253_at | PXK | -2.1 | -1.5 | -1.7 | autophagy-regulatory host factors |
| 1424632_a_at | REV3L | -2.1 | -1.8 | -2.6 | autophagy-regulatory host factors |
| 1426756_at | GALNT2 | -2.4 | -2.0 | -3.0 | autophagy-regulatory host factors |
| 1416536_at | MUM1 | -2.1 | -2.2 | -2.1 | autophagy-regulatory host factors |
| 1419186_a_at | ST8SIA4 | -4.9 | -3.9 | -5.8 | autophagy-regulatory host factors |
| 1442454_at | TOP2A | -5.5 | -4.7 | -3.5 | autophagy-regulatory host factors |
| 1415834_at | DUSP6 | -1.5 | -2.6 | -1.1 | kinases and phosphatases regulatory host factors |
| 1448370_at | ULK1 | -1.3 | -1.1 | -2.7 | kinases and phosphatases regulatory host factors |
| 1456727_a_at | CSNK1D | 1.9 | 1.7 | 2.1 | kinases and phosphatases regulatory host factors |
| 1456844_at | CAMK2D | 3.7 | 3.2 | 10.3 | kinases and phosphatases regulatory host factors |
