# Supplementary material for: Transcriptome Analysis Reveals Novel Entry Mechanisms and a Central Role of SRC in Host Defense during High Multiplicity Mycobacterial Infection
Source: PLoS One. 2013 Jun 18;8(6):e65128. doi: 10.1371/journal.pone.0065128 (PMC3688827; doi:10.1371/journal.pone.0065128)
Supplement: Table S7 — List of the primers used for qPCR. The list represents the list of primer sequences used for SYBR Green quantitative real time PCR (qPCR) (figure 3) with additional primers for 3 housekeeping genes. (DOCX) [file pone.0065128.s007.docx]

| **Index** | **Gene Name** | **Direction** | **Sequence (5' - 3')** |
| --- | --- | --- | --- |
| 1 | 18S | Forward | GAGACTCTGGCATGCTAACTAG |
| 2 | 18S | Reverse | GGACATCTAAGGGCATCACAG |
| 3 | AHR | Forward | AGCRCGGRTGCRAGARAAARCAGRTAA |
| 4 | AHR | Reverse | AGGRCGGRTCTRAACRTCTRGTGRTTC |
| 5 | ATF3 | Forward | GAGRGATRTTTRGCTRAACRCTGRACARCC |
| 6 | ATF3 | Reverse | TTGRACGRGTARACTRGACRTCCRAGC |
| 7 | AURKB | Forward | CAGRAAGRGAGRAACRGCCRTACRCC |
| 8 | AURKB | Reverse | GAGRAGCRAAGRCGCRAGARTGTRC |
| 9 | BCL2A1A | Forward | GGCRTGARGCARCTARCCTRTCARGTA |
| 10 | BCL2A1A | Reverse | TGGRCGGRTATRCTARTGGRATTRCCARC |
| 11 | BETA-ACTIN | Forward | ACGGCCAGGTCATCACTATTG |
| 12 | BETA-ACTIN | Reverse | CAAGAAGGAAGGCTGGAAAAGA |
| 13 | CCL3 | Forward | TTCRTCTRGTARCCARTGARCACRTCTRGC |
| 14 | CCL3 | Reverse | CGTRGGARATCRTTCRCGGRCTGRTAG |
| 15 | CCL4 | Forward | TTCRCTGRCTGRTTTRCTCRTTARCACRCT |
| 16 | CCL4 | Reverse | CTGRTCTRGCCRTCTRTTTRGGTRCAG |
| 17 | CCL5 | Forward | GCTRGCTRTTGRCCTRACCRTCTRCC |
| 18 | CCL5 | Reverse | TCGRAGTRGACRAAARCACRGACRTGC |
| 19 | CCL7 | Forward | GCTRGCTRTTCRAGCRATCRCAARGTG |
| 20 | CCL7 | Reverse | CCARGGGRACARCCGRACTRACTRG |
| 21 | CCND3 | Forward | CGARGCCRTCCRTACRTTCRCAGRTG |
| 22 | CCND3 | Reverse | GGARCAGRGTARGCGRATCRCAGRGT |
| 23 | CENPF | Forward | GCARCAGRCACRAGTRATGRACCRAGG |
| 24 | CENPF | Reverse | CTCRTGCRGTTRCTGRTCGRGTGRAC |
| 25 | CSF1 | Forward | GGCRTTGRGCTRTGGRGATRGATRTCT |
| 26 | CSF1 | Reverse | GAGRGGTRCTGRGCARGGTRACTRC |
| 27 | CXCL10 | Forward | CCARAGTRGCTRGCCRGTCRATTRTTC |
| 28 | CXCL10 | Reverse | GGCRTCGRCAGRGGARTGARTTTRCAA |
| 29 | CXCL2 | Forward | CCARACCRACCRAGGRCTARCAGRG |
| 30 | CXCL2 | Reverse | GCGRTCARCACRTCARAGCRTCTRG |
| 31 | GAPDH | Forward | ACCACAGTCCATGCCATCAC |
| 32 | GAPDH | Reverse | ACCTTGCCCACAGCCTTG |
| 33 | ICAM1 | Forward | GTGRATGRCTCRAGGRTATRCCARTCCRA |
| 34 | ICAM1 | Reverse | CACRAGTRTCTRCAARAGCRACARGCG |
| 35 | IL10 | Forward | GCTRCTTRACTRGACRTGGRCATRGAG |
| 36 | IL10 | Reverse | CGCRAGCRTCTRAGGRAGCRATGRTG |
| 37 | IL1A | Forward | GCARCCTRTACRACCRTACRCAGRAGT |
| 38 | IL1A | Reverse | AAARCTTRCTGRCCTRGACRGAGRCTT |
| 39 | IL1B | Forward | GCARACTRGTTRCCTRGAARCTCRAACRT |
| 40 | IL1B | Reverse | ATCRTTTRTGGRGGTRCCGRTCARACT |
| 41 | IL1RN | Forward | GCTRCATRTGCRTGGRGTARCTTRACARA |
| 42 | IL1RN | Reverse | CCARGACRTTGRGCARCAARGACRAGG |
| 43 | IL6 | Forward | TAGRTCCRTTCRCTARCCCRCAARTTTRCC |
| 44 | IL6 | Reverse | TTGRGTCRCTTRAGCRCACRTCCRTTC |
| 45 | IL6RA | Forward | CCTRGAGRACTRCAARGCARGAARATGRG |
| 46 | IL6RA | Reverse | AGARAGGRAAGRGTCRGGCRTTCRAGT |
| 47 | IRG1 | Forward | AATRGAARACCRTTGRGGTRCTTRATGRCC |
| 48 | IRG1 | Reverse | TGCRCCARTGARCTTRATCRCAGRACARG |
| 49 | ITGA5 | Forward | CTTRCTCRCGTRGGARGTTRTTARCCG |
| 50 | ITGA5 | Reverse | GCTRGTCRAAARTTGRAATRGGTRGGTRG |
| 51 | ITGAV | Forward | CCGRTGGRACTRTCTRTCGRAGCRC |
| 52 | ITGAV | Reverse | CTGRTTGRAATRCAARACTRCAARTGGRGC |
| 53 | JUNB | Forward | TCARCGARCGARCTCRTTARCGCRAG |
| 54 | JUNB | Reverse | CCTRTGARGACRCCCRGATRAGGRGA |
| 55 | KIF11 | Forward | ACCRTCCRAAGRAAARACARTACRACGRTT |
| 56 | KIF11 | Reverse | CGARAGARTGGRTGCRAATRTATRAGCRCC |
| 57 | MAF | Forward | GGARGACRCGARCCGRCATRCATRC |
| 58 | MAF | Reverse | TCARTCCRAGTRAGTRAGTRCTTRCCARGG |
| 59 | NLRP3 | Forward | ATTRACCRCGCRCCGRAGARAAGRG |
| 60 | NLRP3 | Reverse | TCGRCAGRCAARAGARTCCRACARCAG |
| 61 | OSM | Forward | ATGRCAGRACARCGGRCTTRCTARAGA |
| 62 | OSM | Reverse | TTGRGAGRCAGRCCARCGARTTGRG |
| 63 | TNF | Forward | CCCRTCARCACRTCARGATRCATRCTTRCT |
| 64 | TNF | Reverse | GCTRACGRACGRTGGRGCTRACARG |
| 65 | TNFAIP3 | Forward | GAARCAGRCGARTCARGGCRCAGRG |
| 66 | TNFAIP3 | Reverse | GGARCAGRTTGRGGTRGTCRTCARCATRT |
| 67 | TNFRSF1B | Forward | ACARCCCRTACRAAARCCGRGAARCC |
| 68 | TNFRSF1B | Reverse | AGCRCTTRCCTRGTCRATARGTARTTCRCT |
